# Hormone therapy is associated with lower Alzheimer’s disease tau biomarkers in post-menopausal females -evidence from two independent cohorts

**DOI:** 10.1186/s13195-024-01509-5

**Published:** 2024-07-22

**Authors:** Yi-Ting Wang, Joseph Therriault, Cécile Tissot, Stijn Servaes, Nesrine Rahmouni, Arthur Cassa Macedo, Jaime Fernandez-Arias, Sulantha S. Mathotaarachchi, Jenna Stevenson, Firoza Z. Lussier, Andréa L. Benedet, Tharick A. Pascoal, Nicholas J. Ashton, Henrik Zetterberg, Kaj Blennow, Serge Gauthier, Pedro Rosa-Neto

**Affiliations:** 1 Translational Neuroimaging Laboratory, McGill Research Centre for Studies in Aging, Montreal, Canada; 2 Department of Neurology and Neurosurgery, Faculty of Medicine, McGill University, Montreal, Canada; 3 Department of Neurology and Psychiatry, University of Pittsburgh School of Medicine, Pittsburgh, USA; 4 Department of Psychiatry and Neurochemistry, Institute of Neuroscience and Physiology, The Sahlgrenska Academy, University of Gothenburg, Mölndal, Sweden; 5 Centre for Age-Related Medicine, Stavanger University Hospital, Stavanger, Norway; 6 Institute of Psychiatry, Psychology and Neuroscience, Maurice Wohl Institute Clinical Neuroscience Institute, King’s College London, London, UK; 7 NIHR Biomedical Research Centre for Mental Health and Biomedical Research Unit for Dementia at South London and Maudsley NHS Foundation, London, UK; 8 Clinical Neurochemistry Laboratory, Sahlgrenska University Hospital, Mölndal, Sweden; 9 Department of Neurodegenerative Disease, UCL Institute of Neurology, Queen Square, London, UK; 10 UK Dementia Research Institute at UCL, London, UK; 11 Hong Kong Center for Neurodegenerative Diseases, Clear Water Bay, Hong Kong China; 12 Wisconsin Alzheimer’s Disease Research Center, University of Wisconsin School of Medicine and Public Health, University of Wisconsin-Madison, Madison, WI USA; 13 Montreal Neurological Institute, Montreal, QC Canada; 14 The McGill University Research Centre for Studies in Aging, 6875 LaSalle Boulevard, H4H 1R3 Montreal, QC Canada

## Abstract

**Background:**

Females represent approximately 70% of the Alzheimer’s disease (AD) cases and the literature has proposed a connection between the decreased estrogen levels during menopause and an increased AD risk. Previous investigations have predominantly focused on assessing how hormone therapy (HT) affects the likelihood of AD development and cognitive deterioration. However, as the research framework has shifted toward a biomarker-defined AD and alterations in specific biomarkers could take place years before cognitive decline becomes discernible, it is crucial to examine how HT influences AD biomarkers. The main goal of this study was to evaluate the impact of HT on AD biomarker-informed pathophysiology in both cognitively unimpaired (CU) and cognitively impaired (CI) post-menopausal females across the aging and AD spectrum.

**Methods:**

This cross-sectional study included post-menopausal females without HT history (HT-) and with HT (HT+) at the time of PET imaging assessment from two cohorts: the Translational Biomarkers in Aging and Dementia (TRIAD) cohort, and the Alzheimer’s Disease Neuroimaging Initiative (ADNI). Participants underwent magnetic resonance imaging (MRI), positron emission tomography (PET) and biofluid collection. Voxel-based t-tests were performed to assess the differences in amyloid-β (Aβ) and tau neurofibrillary tangles (NFTs) loads between HT- and HT + females. Linear regression models with interaction terms were also conducted to examine the interactive effects of HT and Aβ-PET on regional tau-PET.

**Results:**

HT + females demonstrated significantly lower tau-PET standardized uptake value ratio (SUVR) in Braak I-II ROIs (*P* < 0.05, Hedges’ g = 0.73), Braak III-IV ROIs (*P* < 0.0001, Hedges’ g = 0.74) and Braak V-VI ROIs (*P* < 0.0001, Hedges’ g = 0.69) compared to HT- females. HT + females also showed significantly lower CSF p-tau_181_ (*P* < 0.001) and plasma p-tau_181_ (*P* < 0.0001) concentrations. Additionally, results from multivariate linear regression models indicated that HT interacts with cortical Aβ and is associated with lower regional NFT load.

**Conclusions:**

Overall, findings from this observational study suggest that HT is associated with lower tau neuroimaging and fluid biomarkers in postmenopausal females. Due to the close link between tau and cognition, this study highlights the need for large randomized controlled trials designed to systemically study the influences of HT on AD biomarkers and disease progression.

**Supplementary Information:**

The online version contains supplementary material available at 10.1186/s13195-024-01509-5.

## Introduction

Alzheimer’s disease (AD) is the most common form of dementia and is characterized by the accumulation of two pathological protein aggregates: amyloid-β (Aβ) plaques and tau neurofibrillary tangles (NFTs) [1]. Female makes up almost two-thirds of the AD population worldwide [2, 3]. The higher age-standardized dementia prevalence in females (female-to-male ratio = 1.69 (1.64–1.73)) shown in the recent 2022 Global Burden of Disease (GBD) report provided evidence that higher incident cases in females cannot simply be explained by greater life expectancy [4]. Findings from preclinical and clinical studies also supported the sex-specific biological mechanisms in diverging AD risk as an important adjunct explanation to the epidemiologic perspective. More specifically, the neurophysiological impact of estrogen decline is emerging as the main aetiological basis for the higher prevalence of AD in females [5–7]. Evidence from positron emission tomography (PET) imaging studies demonstrated that peri/post-menopausal females exhibit increased brain Aβ deposition as compared to premenopausal females and age-matched males [8, 9]. Results from animal studies discovered that estrogen receptor α co-localized with NFTs and estradiol seemed to show a protective effect against tau hyperphosphorylation, particularly among female rats [10]. A recent imaging study suggested that earlier age at menopause was associated with increased tau vulnerability especially when neocortical Aβ was elevated [11]. In line with this, surgically-induced menopause was also implicated to be associated with more prominent AD neuropathology [12–14].

In recent decades, multiple research groups started to investigate the effects of administrating hormone therapy (HT) as a preventive strategy against AD risk and long-term change in cognitive function in perimenopausal and postmenopausal females, yet the results have been inconclusive [15–18]. Early meta-analyses of observational studies on the relationship between AD and HT use/history suggested significant reductions between 29% and 44% in the risk of AD for women who used HT in their lifetime versus those who had never used HT [15, 19–22]. These earlier findings, however, were called into question after the publication of data from the Women’s Health Initiative Memory Study (WHIMS), which revealed that HT was not only not beneficial for the prevention of dementia but may also increase the risk for cognitive decline and dementia in women over age 65 [23–26]. Findings from the ancillary Cognitive and Affective Study (KEEPS-Cog) of the Kronos Early Estrogen Prevention Study (KEEPS) also showed no alteration in cognition in recently postmenopausal women with menopausal HT (MHT, administered proximal to the menopausal transition) [27]. Another large, randomized control trial, the Early versus Late Intervention Trial with Estradiol (ELITE) trials, utilizes oral estradiol delivered for up to 5 years to participants whose menopause transition was remote (10 years beyond menopause) or recent, to elucidate appropriate timing or the window of opportunity to initiate HT [28]. Results from a recent meta-analysis examining 6 random controlled trials and 45 observational reports suggest that estrogen therapy initiated during the critical window of the menopause transition may reduce the risk of developing AD [19]. Overall, the current consensus is that HT administered well past the menopausal transition will not prevent AD and may even elevate risk, especially if the individual is already exhibiting preclinical or subclinical neurodegenerative changes or metabolic dysregulation. While MHT might hold potential as an AD prevention strategy, it remains to be determined precisely what subgroups of women could benefit from HT and for whom HT is contraindicated.

Previous investigations have predominantly concentrated on assessing how HT affects the likelihood of AD development and cognitive deterioration, neglecting to explore the impacts of HT on AD-related biomarkers. As the research framework has shifted toward a biomarker-defined AD in living persons [29], and alterations in specific AD biomarkers could take place years before cognitive decline becomes discernible, it is crucial to examine how HT influences the levels of AD biomarkers. Therefore, the main goal of this study was to evaluate the impact of estrogen-based HT on AD biomarker-informed pathophysiology to elucidate the crosslinks between HT, Aβ and tau in post-menopausal females.

## Materials and methods

### Participants

#### Translational biomarkers in aging and dementia (TRIAD)

TRIAD is an ongoing longitudinal study launched in 2017 at the McGill Centre for Studies in Aging. In this study, we assessed a total of 201 female participants from the TRIAD cohort including 178 post-menopausal HT non-users (HT-) and 23 post-menopausal HT users (HT+). All participants underwent structural MRI, Aβ-PET with [^18^F]AZD4694, and tau-PET with [^18^F]MK6240. Cerebrospinal fluid (CSF) and plasma were also collected for some participants (CSF: *n* = 55 and plasma: *n* = 107). All participants additionally underwent clinical and cognitive assessments, including the Mini-Mental State Examination (MMSE), Montreal Cognitive Assessment (MoCA), Logical Memory Test, Rey Auditory Verbal Learning Test (RAVLT) and the Clinical Dementia Rating (CDR). Objective cognitive impairment is defined as deficits in one or more cognitive domains. On the other hand, subjective cognitive impairment is the self-reported experience of worsening or more frequent confusion or memory loss. In this study, cognitively unimpaired (CU) individuals had a CDR score of 0 and MMSE ≥ 27. Subjects with mild cognitive impairment (MCI) had a CDR score of 0.5 and essentially normal activities of daily living with or without subjective cognitive impairment. Patients with mild-to-moderate sporadic AD dementia met the National Institute on Aging and Alzheimer’s Association criteria for probable AD as determined by a physician and had a CDR score between 0.5 and 2. We excluded participants with inadequately treated systemic conditions, active substance abuse, recent head trauma, recent major surgery or presenting with MRI/PET safety contraindications. The study was approved by the Montreal Neurological Institute PET Working Committee and the Douglas Mental Health University Institute Research Ethics Board. Written informed consent was obtained from all participants.

#### Alzheimer’s disease neuroimaging initiative (ADNI)

In this study, to enhance the reproducibility of our findings, we also incorporated participants from the ADNI cohort. ADNI was launched in 2003 as a public-private partnership, led by Principal Investigator Michael W. Weiner, MD. The primary goal of ADNI has been to test whether serial MRI, PET, other biological markers, and clinical and neuropsychological assessment can be combined to measure the progression of MCI and early Alzheimer’s disease. Analyses were conducted independently in two sub-cohorts: ADNI imaging (*n* = 343, for PET imaging analysis) and ADNI fluid (*n* = 396, for fluid biomarker analysis). The reason for having two sub-cohorts in this study was the fact that only ADNI3 participants underwent tau-PET. By having the two sub-cohort study designs, we could maximize the enrollment of HT + subjects. All participants in the ADNI imaging sub-cohort underwent structural MRI, Aβ-PET with [^18^F]florbetapir, and tau-PET with [^18^F]flortaucipir. All participants in the ADNI fluid sub-cohort had Aβ-PET with [^18^F]florbetapir, and CSF and plasma p-tau measures at the same visit. Data used in the preparation of this article were obtained from the ADNI database. The ADNI study was approved by the institutional review boards of all the participating institutions. Informed written consent was obtained from all participants at each site. Full information regarding the inclusion and exclusion criteria in ADNI can be accessed at http://adni.loni.usc.edu/. There was no attempt to match cases between the ADNI and TRIAD cohorts.

### Hormone therapy assignment

During each visit, participants were asked to provide a list of their current medication prescribed by their doctor(s) specifying information including medication name, dosage, frequency, reason for taking the medication and start date (end date if applicable). The investigators examined the lists to assign hormone therapy status (HT- or HT+) based on the FDA-approved HT medication (https://www.fda.gov/media/119387/download?attachment). HT + females are defined as individuals who were current or previous users of estrogen alone or combination (estrogen plus progestin) HT, while HT- females are those with no record of HT use. Information about initiation age of the HT, route of administration, type and dosage of medications were recorded in both ADNI and TRIAD cohorts, but not examined in this study due to the small sample size. Female individuals with oophorectomy were excluded from this study.

### Brain imaging methodology

#### TRIAD

[^18^F]AZD4694 PET and [^18^F]MK6240 PET scans in the TRIAD cohort were acquired with a brain-dedicated Siemens High-Resolution Research Tomograph (HRRT). [^18^F]AZD4694 images were acquired at 40–70 min after the intravenous bolus injection of the tracer and reconstructed with an ordered subset expectation maximization (OSEM) algorithm on a four-dimensional (4D) volume with 3 frames (3 × 600 s). [^18^F]MK-6240 images were acquired at 90–110 min after the intravenous bolus injection of the tracer and reconstructed using the same OSEM algorithm on a 4D volume with 4 frames (4 × 300 s) [30]. At the end of each PET emission acquisition, a 6-min transmission scan with a rotating ^137^Cs point source was performed for attenuation correction. PET images were also corrected for motion, dead time, decay and scattered and random coincidences. Briefly, PET images were linearly registered to the native T1-weighted MRI and MRIs were linearly and nonlinearly registered to the ADNI standardized space. Then, PET images in the T1 space were brought to the ADNI standardized space using transformations from native MRI to the ADNI standardized space. PET images were subsequently spatially smoothed to an 8-mm full-width at half maximum resolution. [^18^F]AZD4694 standardized uptake value ratio (SUVR) used the whole cerebellum gray matter as the reference region whereas [^18^F]MK6240 SUVRs used the inferior cerebellar gray matter. Global [^18^F]AZD4694 SUVR value was estimated for each participant by averaging the SUVR from the precuneus, prefrontal, orbitofrontal, parietal, temporal, anterior and posterior cingulate cortices. Regional [^18^F]MK6240 SUVRs were generated for meta-ROIs including the entorhinal, amygdala, parahippocampal, fusiform, inferior temporal and medial temporal regions. Aβ positivity was assigned based on published cut-offs of [^18^F]AZD4694 neocortical SUVR (1.55 SUVR) [31].

#### ADNI

Full information regarding the acquisition and pre-processing of PET data in ADNI is provided at http://adni.loni.usc.edu/data-samples/pet/. Pre-processed PET images downloaded from ADNI underwent spatial normalization to the ADNI standardized space using the transformations of PET native to MRI native space and MRI native to the ADNI space. [^18^F]florbetapir SUVR and [^18^F]flortaucipir SUVR were generated using the whole cerebellar grey matter and the inferior cerebellar grey matter as reference region, respectively. A global [^18^F]florbetapir SUVR value was estimated for each participant by averaging the SUVR from the precuneus, prefrontal, orbitofrontal, parietal, temporal, anterior and posterior cingulate cortices. Regional [^18^F]flortaucipir SUVRs were generated for each Braak staging ROI as well as meta ROIs.

### Fluid biomarker measurements

CSF and plasma collection in the TRIAD cohort followed procedures previously described [32]. All measures were quantified at the University of Gothenburg (Gothenburg, Sweden), by scientists blinded to the clinical and biomarker data. CSF concentrations of p-tau_181_ and p-tau_217_ were quantified using a custom Single molecular array (Simoa) assay as described previously [33]. Plasma p-tau_181_ was measured by in-house Simoa methods on an HD-X Analyzer (Quanterix, Billerica, MA, USA) [34]. Plasma p-tau_217_ was measured on the Simoa machine using Janssen R&D assay [35]. Detailed information is described in Supplementary Method.

### Neuroimaging voxel-based analyses

Neuroimaging voxel-based analyses were performed using the VoxelStats toolbox (https://github.com/sulantha2006/VoxelStats) in MATLAB R2015a (The MathWorks, Natick, MA, USA, http://www.mathworks.com). VoxelStats is a MATLAB-based analytical framework that allows for the execution of multimodal voxelwise neuroimaging analyses. Welch’s t-test was performed at the voxel level to assess the differences in Aβ and NFT loads between HT- and HT + females. BrainNet Viewer [36] was used for visualization of the results from the neuroimaging analyses.

### Statistical analysis

Statistical analyses were performed in Python 3.9.12 and MATLAB. Demographic (age, years of education) and clinical data (MMSE, concentration of plasma and CSF biomarkers, PET SUVRs) were compared between HT- and HT + females using independent *t-*tests or Welch’s t-tests (accounting for unequal sample sizes and unequal variances) with Bonferroni correction, as appropriate. Categorical variables (*APOEε4* carriage status, clinical diagnosis) were compared using the χ2 test. CU and CI (cognitively impaired, including both MCI and AD dementia diagnosis) individuals are previously defined in the Participant section. Linear regression models were fitted with an interaction term to estimate the extent to which HT use moderated the association between cortical Aβ load and regional tau aggregation. The regression models were adjusted for age, education, *APOE* carriage status and clinical diagnosis (CU or CI) to account for their potential influence.

## Results

### Participants

Demographic characteristics of the study populations are displayed in Table 1. Of 201 female individuals in the TRIAD cohort, 23 were HT+ (11.4%) and 178 were HT- (88.6%). In the ADNI cohort (ADNI Imaging: *n* = 343 and ADNI Fluid: *n* = 396), 75 were HT+ (10.1%) and 664 were HT- (89.9%). Briefly, HT + females showed lower regional tau-PET and lower CSF and plasma p-tau concentrations. They performed better in the MMSE test compared to HT- individuals. No significant difference was reported regarding the educational attainment and *APOE* genotypes between HT- and HT + groups.

**Table 1:** Demographics of the study populations

	TRIAD cohort		ADNI imaging cohort		ADNI fluid cohort	
	HT-	HT+	HT-	HT+	HT-	HT+
No.	178	23	315	28	349	47
Age, mean (SD), y	71.4 (6.7)	73.1 (5.8)	72.8 (8.6)	71.5 (7.9)	73.0 (7.4)	70.5 (6.4) *
Education, mean (SD), y	14.8 (3.6)	15 (3.2)	16.1 (2.4)	16.5 (2.2)	15.6 (2.6)	16.3 (2.5)
Clinical Diagnosis, CU: CI	97: 81	16: 7	187: 128	16: 12	123: 226	23: 24
*APOE ε4* carrier (%)	33.7%	27.3%	41.6%	40%	46.4%	34%
MMSE score, mean (SD)	26.9 (4.9)	29.2 (1.0) *	27.8 (3.6)	28.1 (3.3)	27.3 (3.2)	28.7 (1.6) *
RAVLT score ^†^						
Recognition, mean (SD)	10.0 (6.9)	12.1 (3.4) *				
Immediate, mean (SD)			44.2 (12.3)	43.9 (12.7)	39.2 (13.2)	47.8 (11.8) *
Learning, mean (SD)			5.7 (2.7)	5.5 (2.6)	4.7 (2.9)	6.0 (2.8) *
Forgetting, mean (SD)			3.9 (3.0)	4.0 (3.2)	4.5 (2.7)	3.8 (2.7)
**Alzheimer’s disease biomarkers**						
Amyloid-PET Neocortical SUVR	1.87 (0.67)	1.77 (0.56)	1.19 (0.24)	1.12 (0.17)	1.24 (0.24)	1.16 (0.23) *
Tau-PET imaging biomarkers					Tau fluid biomarkers	
Braak I-II SUVR	1.24 (0.54)	1.05 (0.26) *	1.22 (0.15)	1.24 (0.15)	CSF p-tau_181_	
Braak III-IV SUVR	1.35 (0.88)	0.98 (0.19) *	1.17 (0.17)	1.14 (0.11) *	29.09 (16.3)	22.92 (10.2) *
Braak V-VI SUVR	1.25 (0.79)	0.91 (0.13) *	1.05 (0.14)	1.01 (0.11) *	Plasma p-tau_181_	
META-ROI SUVR	1.39 (0.94)	0.99 (0.26) *	1.22 (0.20)	1.18 (0.13) *	18.59 (10.1)	13.44 (6.9) *
**Hormone Therapy Information**						
Status (Past: New: Current) ^††^		0: 1: 22		0: 7: 21		3: 6: 38
Type (Estrogen only: Combination)		14: 9		23: 5		42: 5
Starting age, mean (SD), y		55.4 (11.1)		60.6 (12.6)		59.9 (11.2)
Duration, mean (SD), y		16.8 (11.7)		10.1 (9.6)		10.5 (10.8)

Variables including age, education level, MMSE score, AD neuroimaging and fluid biomarker levels were assessed using independent t-tests, or Welch’s t-tests (when the group variance reported significantly different), to evaluate if significant differences exist between HT- and HT + groups. Categorical variables including *APOEε4* carriage status and clinical diagnosis were compared using the χ2 test. Overall, we reported no significant difference between HT- and HT + regarding educational attainment, *APOEε4* carriage status and clinical diagnosis. HT + females in the TRIAD cohort and the ADNI Imaging cohort showed lower regional tau-PET SUVRs compared to HT- females. HT + females in the ADNI fluid cohort demonstrated lower concentrations of p-tau in both CSF and plasma

^*^Significantly different

^†^ Different summary scores are derived from raw RAVLT scores. These include Recognition (the sum of recognition minus false positives), Immediate (the sum of scores from 5 first trials, i.e., Trials 1 to 5), Learning (the score of Trial 5 minus the score of Trial 1) and Forgetting (the score of Trial 5 minus the score of the delayed recall)

^††^ HT status: “New” indicated an individual using HT for less than a year at the time of the PET imaging assessment

*Abbreviation*: HT: hormone therapy; CU: cognitively unimpaired; CI: cognitively impaired; MMSE: Mini-mental state examination; RAVLT: Rey Auditory Verbal Learning Test; SUVR: standardized uptake value ratio

### Hormone therapy mitigates tangle aggregation and tau phosphorylation in post-menopausal females

**Fig. 1:**
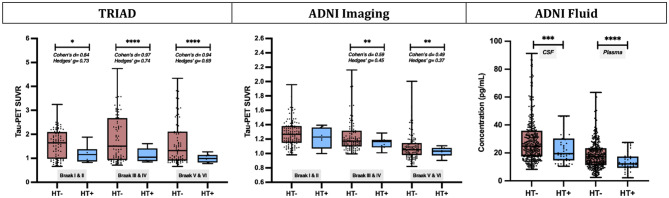
Hormone therapy mitigates NFT load and p-tau concentrations in post-menopausal females. HT+ females demonstrated significantly lower tau-PET SUVRs in Braak I-II ROIs (TRIAD: P < 0.05, Hedges’ g = 0.73), Braak III-IV ROIs (TRIAD: P < 0.0001, Hedges’ g = 0.74; ADNI: P < 0.01, Hedges’ g = 0.45) and Braak V-VI ROIs (TRIAD: P < 0.0001, Hedges’ g = 0.69; ADNI: P < 0.01, Hedges’ g = 0.37) compared to HT- females. HT + females also showed significantly lower CSF p-tau_181_ (P < 0.001) and plasma p-tau_181_ (P < 0.0001) concentrations

Results from independent t-tests showed that HT + females demonstrated lower regional tau-PET SUVR (Fig. 1). In the TRIAD cohort, the HT + group presented significantly lower tau-PET SUVR in Braak I-II ROIs (*P* < 0.05, Hedges’ g = 0.73), Braak III-IV ROIs (*P* < 0.0001, Hedges’ g = 0.74) and Braak V-VI ROIs (*P* < 0.0001, Hedges’ g = 0.69). We found consistent results in the ADNI cohort where HT + females showed lower tau-PET SUVR in Braak III-IV ROIs (*P* < 0.01, Hedges’ g = 0.45) and Braak V-VI ROIs (*P* < 0.01, Hedges’ g = 0.37). Additionally, results from the ADNI Fluid cohort suggested that HT + females also had significantly lower CSF p-tau_181_ (*P* < 0.001) and plasma p-tau_181_ (*P* < 0.0001) concentrations.

### HT + post-menopausal females presented lower tau tangle load in the brain

**Fig. 2:**
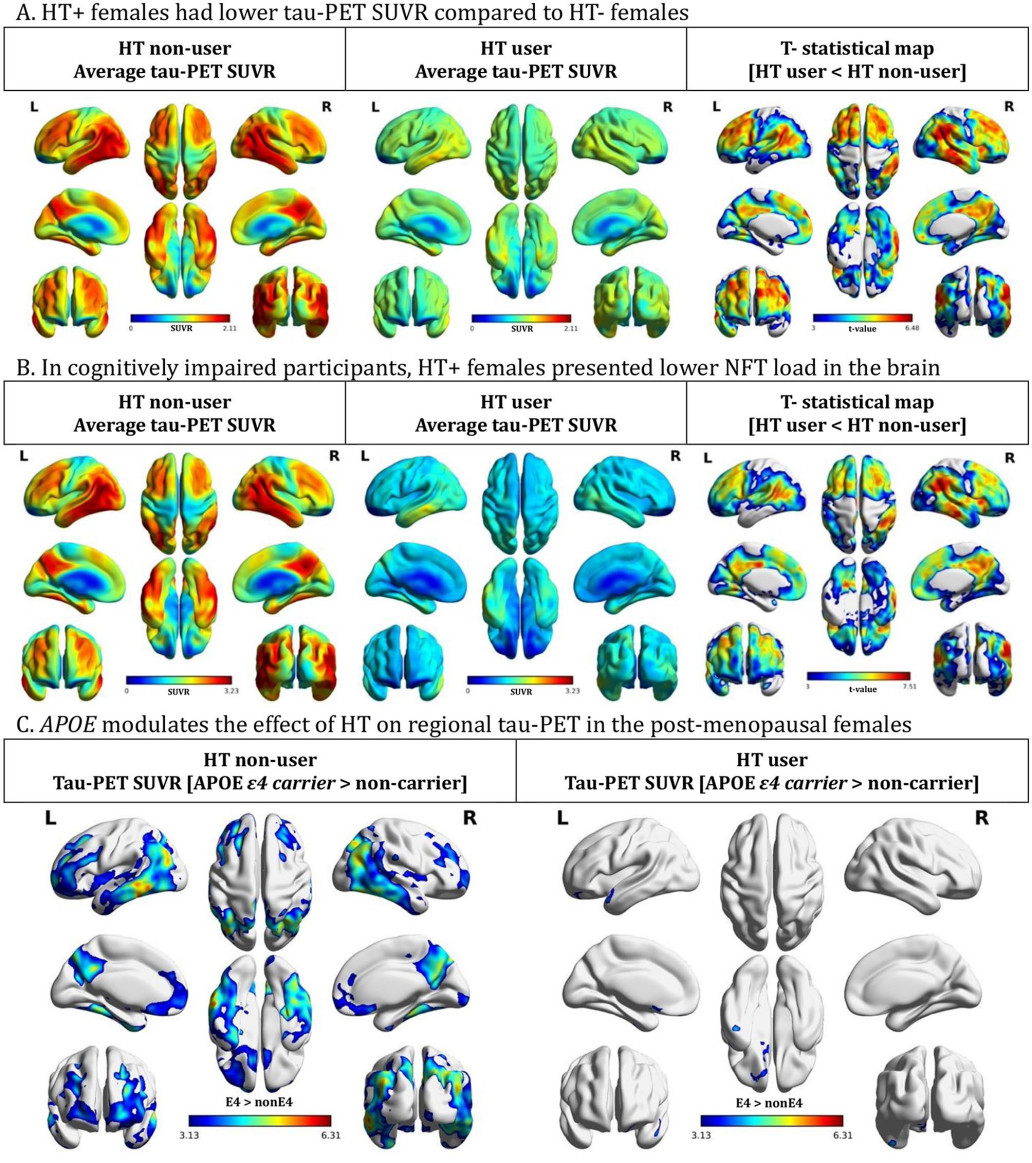
Hormone therapy use is linked to lower regional tau load in post-menopausal females. (**A**) Results from voxel-based Welch’s t-test showed that HT + females presented significantly lower tau-PET SUVR in multiple brain regions as demonstrated in the t-statistical map. (**B**) Among cognitively impaired subjects, HT + females also demonstrated significantly less NFT load compared to HT- females. (**C**) In post-menopausal HT non-users, *APOEε4* carriers presented with significantly higher NFT load compared to *APOEε4* non-carriers. In contrast, post-menopausal females who use HT showed similar levels of tau-PET signals, regardless of their APOE genotypes. Images represent voxel-based t-statistical parametric maps overlaid on the structural MRI reference template. Results were corrected for multiple comparisons using the FDR cluster threshold of *P* < 0.001

We then compared the NFT load in the brains of HT- and HT + post-menopausal females. Welch’s t-test was performed at the voxel level, and we found that HT + females presented significantly less NFT load in the brain as compared to HT- females (Fig. 2A). Among cognitively impaired (CI) subjects, HT + females again demonstrated lower tau-PET SUVR compared to HT- females (Fig. 2B). Additionally, our results indicated that *APOE* modulates the effect of HT on regional Aβ-PET and tau-PET. In post-menopausal HT non-users, *APOEε4* carriers presented with significantly higher Aβ and NFT load compared to *APOEε4* non-carriers. In contrast, post-menopausal females who use HT showed similar levels of Aβ-PET and tau-PET, regardless of their APOE genotypes (Fig. 2C and Supplementary Fig. 1).

### Hormone therapy interacts with cortical Aβ and is associated with lower regional NFT load

**Fig. 3:**
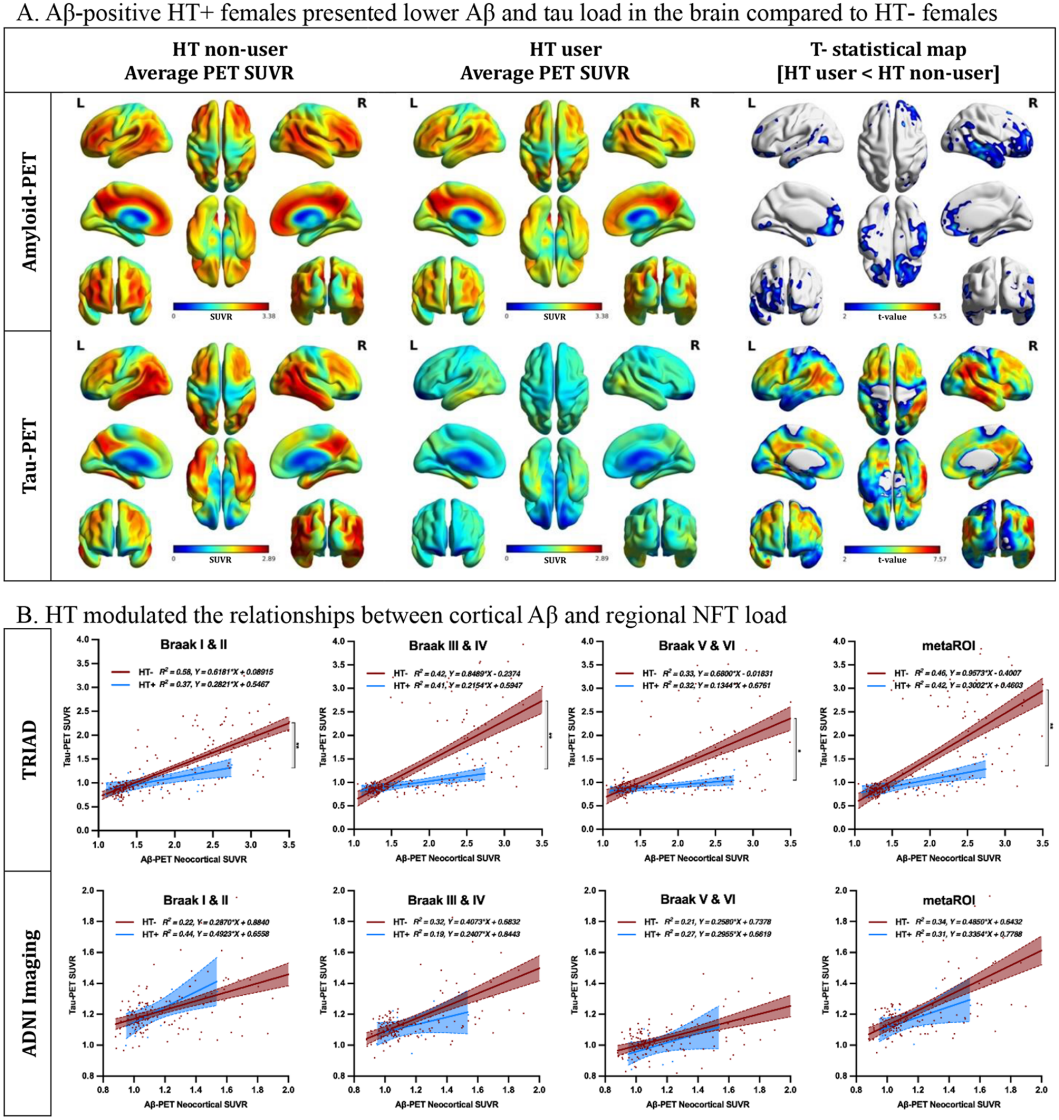
Hormone therapy interacts with cortical Aβ and is associated with lower regional NFT load. (**A**) We assessed how HT affected the average Aβ and NFT load in participants with prominent Aβ pathology (Aβ+ subjects). Voxel-based Welch’s t-test showed that HT + females presented significantly lower Aβ-PET SUVR in temporal and frontal regions and had significantly lower tau-PET SUVR in multiple brain regions compared to HT- females. (**B**) Linear regression models showed that with similar Aβ load, HT + females demonstrated less NFT aggregation compared to HT- females, suggesting HT use interacted with cortical Aβ and mitigated regional NFT load

We next performed voxel-based Welch’s t-tests to compare the average Aβ and NFT load in participants with prominent Aβ pathology (Aβ + subjects). We observed that HT + females presented significantly lower Aβ-PET SUVR in temporal and frontal regions; they also had lower tau-PET SUVR in multiple brain areas (Fig. 3A). Importantly, with similar Aβ-PET SUVRs, HT + females displayed lower tau-PET SUVR in Braak ROIs and meta-ROIs (Fig. 3B and Supplementary Fig. 2). Additionally, HT + females also presented lower p-tau concentrations compared to HT- females (Supplementary Fig. 3). To elucidate the cross-links between HT, Aβ and tau, linear regression models with interaction terms were conducted. The findings indicated that HT interacted with Aβ and was associated with lower regional tau-PET (Table 2). The results remained significant after correcting for age, education, *APOE* genotypes and clinical diagnosis.

**Table 2:**
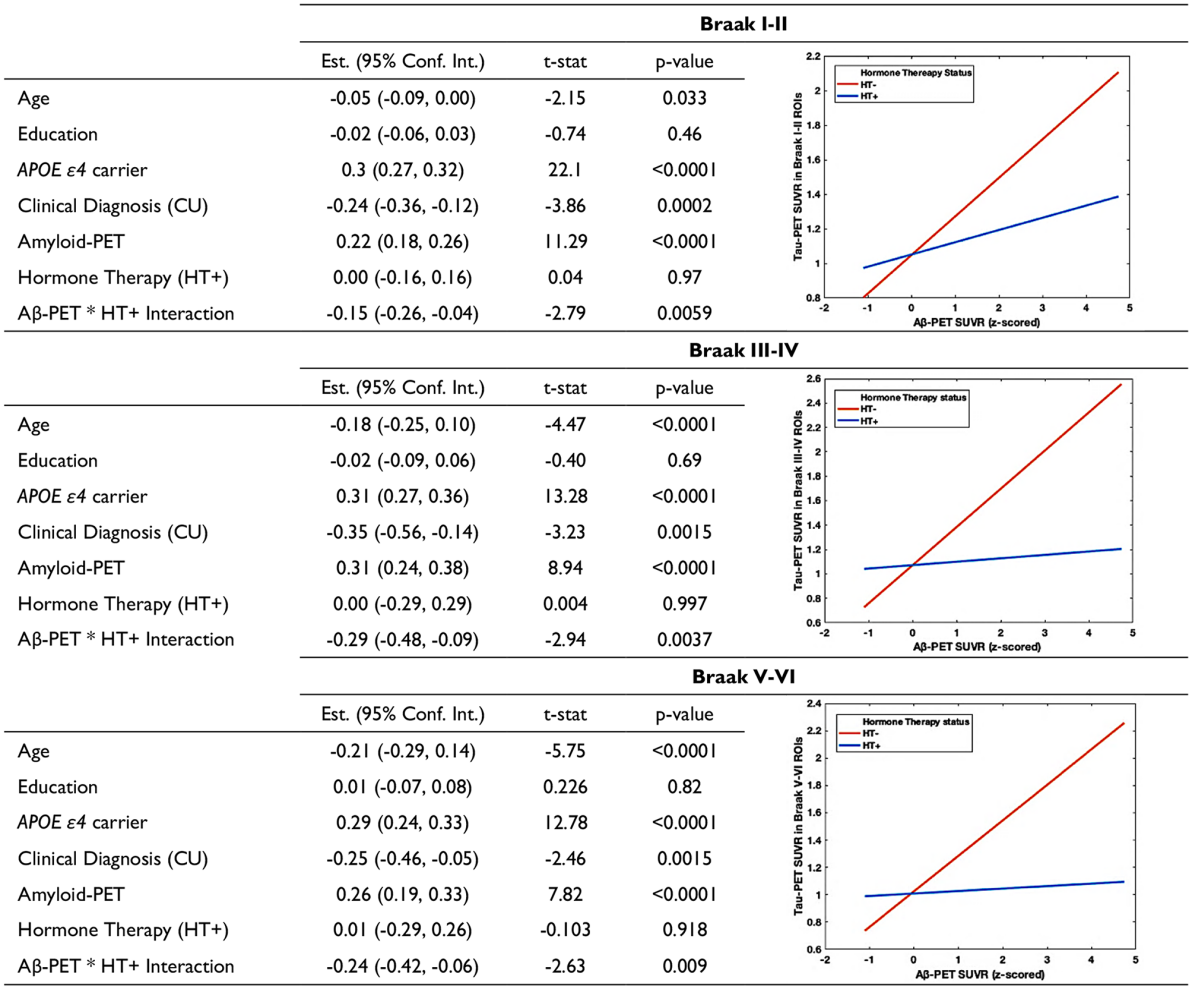
Hormone therapy interacts with cortical Aβ and is associated with lower regional tau-PET SUVRs

Multivariate linear regression models were performed to understand how the interaction between Aβ and HT influenced regional tau load in the brain. The results showed that HT interacted with neocortical Aβ-PET and is associated with lower tau-PET in Braak ROIs. The findings remained significant after correcting for age, education, *APOE* ε*4* carriage status and clinical diagnosis. Interaction plots are demonstrated on the right side of the table

Abbreviation: HT: hormone therapy; CU: cognitively unimpaired

## Discussion

The higher risk of AD in females highlights the need for sex-specific investigations into the pathogenesis of AD. The decline in estrogen levels during menopause has been indicated as a contributing factor to the pathological progression of AD in females. Despite extensive research efforts to investigate the impact of HT on AD risk and long-term cognitive change, results of administrating HT in perimenopausal and postmenopausal females as a preventive strategy against AD have been inconclusive [15–18]. Previous investigations have predominantly focused on assessing how HT affects the likelihood of AD development and cognitive deterioration, neglecting to explore the influence of HT on AD-related biomarkers. As the research framework has shifted toward a biomarker-defined AD [29], it is crucial to examine how HT influences the levels of AD biomarkers. In this present observational study, we evaluated the relationships between HT use and AD biomarker-informed pathophysiology to understand the crosslinks between HT and two AD primary pathological hallmarks, Aβ and tau. We reported that HT is associated with lower tau neuroimaging and fluid biomarkers in post-menopausal females. Taken together, findings from this study highlight the need for large randomized controlled trials designed to comprehensively study the influence of HT on AD biomarkers and progression in middle-aged females.

Recent findings have identified *APOE* genotype and age of HT initiation as potential modulators of the effect of HT intervention [11, 37, 38]. *APOEε4* has been known to be the most important genetic risk factor for sporadic AD. A greater penetrance of an *APOE*ε*4* genotype in females was suggested to be an important contributor to the higher AD rates in women [39]. Indeed, multiple studies have reported a sex-imposed deleterious effect of *APOEε4*. Female *APOEε4* carriers were found to have worse episodic memory [40], lower default-mode network activity [41], decreased hippocampal connectivity [42] and increased hypometabolism and atrophy [43] in comparison to age-matched male *APOEε4* carriers. One meta-analysis showed a stronger association between *APOEε4* and higher CSF tau burden among women compared with men, and this association was only observed in individuals with evident Aβ pathology [44]. Another study also demonstrated that in cognitively normal older adults, females had more tau tangles in the entorhinal cortex than males, and this sex difference was slightly more pronounced in *APOEε4* carriers[45]. In line with this, our previous study also found that female *APOEε4* carriers presented significantly higher NFT burden in early tau deposition regions including the hippocampus, entorhinal and parahippocampal cortices compared to male *APOEε4* carriers [46]. Interestingly, the estrogen receptor α appeared to be responsible for the estrogen-mediated upregulation of *APOE* expression [47], indicating that estrogen and *APOE* might act synergistically in postmenopausal females. Although the available evidence remained inconclusive regarding the role *APOEε4* plays in modulating the effect of HT on AD-related pathologies, results from voxel-based analyses in this study revealed that among the post-menopausal HT + females, *APOEε4* carriers showed similar level of Aβ-PET and tau-PET as *APOEε4* non-carriers. In contrast, in the post-menopausal HT- group, *APOEε4* carriers presented with significantly higher Aβ and NFT load compared to *APOEε4* non-carriers (Fig. 2C and Supplementary Fig. 1). Our findings are in agreement with two other studies that reported *APOEε4* females received favourable outcomes (A*β* pathway biomarker level [48], improved cognition and larger brain volumes [37]) from HT.

Besides the *APOE* genotype, the timing of HT initiation has also been indicated as a mediator of the cognitive impact of HT use, leading to the critical window hypothesis [49, 50]. This hypothesis suggests that the neuroprotective effects of HT are only evident when it is introduced during the menopausal transition or early post-menopausal period, where gradual estrogen decline increases the brain’s liability to AD-related pathologies. In 2004, WHIMS published results suggesting the use of estrogen plus progestin HT to prevent the incidence of MCI and dementia is not recommended in women 65 years of age or older [23]. WHIMS answered critically important questions about whether HT can protect against dementia in elderly women who start HT years after menopause. However, the critical window hypothesis has prompted us to question the generalisability of WHIMS to perimenopausal females experiencing menopausal symptoms, for whom HT is considered appropriate shortly after menopause. Observational studies that examined the timing of initiation of HT in relation to AD risk [43–45] and cognitive test performance [46–48] both support the critical window hypothesis. Some randomized clinical trials of estrogen therapy in younger women also find support for the hypothesis [49, 50]. However, this notion is challenged by recent results from a Danish nationwide nested case-control study, which indicated a contrary trend. In that study, the use of HT was found to be positively associated with the development of all-cause dementia including AD, even in females who received treatment at the age of 55 years or younger [51]. Nevertheless, the authors did acknowledge the need for additional research to ascertain whether these findings represent an actual effect of HT on dementia risk, or if they signify an underlying susceptibility to dementia among women requiring HT. On the AD biomarker level, HT has been found to be beneficial if introduced before a certain threshold of neuronal damage accumulates, with potentially a critical window where HT can be neuroprotective [11, 52]. In a study analyzing data from UK BIOBANK, despite showing that cumulative lifetime estrogen exposure was associated with increased brain aging, a subgroup analysis revealed that women who started HT earlier had less apparent brain aging compared to later starters. Importantly, this effect of HT timing was only evident in *APOEε4* carriers [53], again raising the notion that interaction between *APOEε4* and HT might have a significant effect on brain health later in life.

### Limitation

This was a cross-sectional observational study precluding the establishment of a causal relationship. Data about the age of menopause, or if there was a gap between age at menopause and the start of HT is not available, which would allow further granularity in our analysis. A further limitation is the small number of participants in the HT + group (10.4%, 98 out of 940 participants). It has been well documented that since the initial publication of WHI in 2002, HT use has decreased substantially [54, 55]. A report published in 2012 showed that in 1999–2000, the prevalence of oral HT use was 22.4% (95% confidence interval [CI] 19.0-25.8) overall, 13.3% (95% CI 11.0-15.5) for estrogen only, and 8.3% (95% CI 6.2–10.4) for estrogen plus progestin. A sharp decline in the use of all formulations occurred between 2003 and 2004, when the overall prevalence decreased to 11.9% (95% CI 9.6–14.2). This decline was initially limited to non-Hispanic whites; use among non-Hispanic blacks and Hispanics did not decline substantially until 2005–2006. Hormone use continued to decline through 2009–2010 across all demographic groups, ending up at approximately 4.7% in 2012 [56]. As TRIAD and ADNI cohorts were both initiated after 2003, inevitably, the generational trends in HT post WHI are leading to the low HT + sample size. This relatively small number led to lower statistical power concerning the analyses performed in the HT + group compared to the HT- group, and also hindered the stratification according to important variables such as *APOEε4* carriage status, the age of HT initiation, the duration of HT, or the use of estrogen-only or combination HT for further investigation. Moreover, the types of estrogen in the HT formulation, the doses and frequency of HT use, and the route of administration were not investigated in this study either. Finally, the findings from this study should not be interpreted as recommending toward using HT as a therapeutical strategy against AD. We recognize and emphasize the inherent limitations of investigating observational data and we would like to highlight the importance of large randomized controlled trials designed to comprehensively study the influence of HT on AD biomarkers and disease progression to fortify the findings reported in this study. Additionally, alterations in specific AD biomarkers take place years before cognitive decline becomes discernible. Therefore, for future clinical trials examining the impact of HT, it is essential to consider the concurrent investigation of both AD biomarkers and cognitive symptoms.

## Conclusion

Findings from the present study support the framework proposing that HT influences AD biomarker-informed tau pathology in post-menopausal females. Considering the tight connection between tau pathology and clinical symptoms, this study highlights the urgent need for new large randomized controlled trials designed to comprehensively study the influence of HT on AD biomarkers and disease progression in middle-aged females.

### Electronic supplementary material

Below is the link to the electronic supplementary material.

Supplementary Material 1

## Data Availability

Data from the TRIAD cohort that support the findings of this study are available from the corresponding author upon reasonable request. All requests for raw and analyzed data and materials will be promptly reviewed by McGill University to verify if the request is subject to any intellectual property or confidentiality obligations. Anonymized data will be shared upon request from a qualified academic investigator for the purpose of replicating the procedures and results presented in this article. Any data and materials that can be shared will be released via a material transfer agreement. Data are not publicly available due to information that could compromise the privacy of research participants.
