# Supplementary material for: Hormone therapy is associated with lower Alzheimer’s disease tau biomarkers in post-menopausal females -evidence from two independent cohorts
Source: Alzheimers Res Ther. 2024 Jul 22;16:162. doi: 10.1186/s13195-024-01509-5 (PMC11265084; doi:10.1186/s13195-024-01509-5)
Supplement: Supplementary file 1 — Supplementary Material 1 [file 13195_2024_1509_MOESM1_ESM.docx]

**Supplementary**

**Hormone therapy is associated with lower Alzheimer’s disease tau biomarkers in post-menopausal females -Evidence from two independent cohorts**

Yi-Ting Wang MSc^1,2^, Joseph Therriault PhD^1,2^, Cécile Tissot PhD^1,2^, Stijn Servaes PhD^1,2^, Nesrine Rahmouni BSc^1,2^, Arthur Cassa Macedo MD^1,2^, Jaime Fernandez-Arias MSc^1,2^, Sulantha S. Mathotaarachchi MSc^1,2^, Jenna Stevenson BA^1,2^, Firoza Lussier MSc^3^, Andréa L. Benedet PhD^4^, Tharick A. Pascoal MD PhD^3^, Nicholas J. Ashton PhD^4,5,6,7^, Henrik Zetterberg MD PhD^4,8,9,10,11,12^, Maria Natasha Rajah PhD ^13^, Kaj Blennow MD PhD^4,8^ and Pedro Rosa-Neto MD, PhD^1,2^

^1^ Translational Neuroimaging Laboratory, McGill Research Centre for Studies in Aging

^2^ Department of Neurology and Neurosurgery, Faculty of Medicine, McGill University

^3^ Department of Neurology and Psychiatry, University of Pittsburgh School of Medicine, Pittsburgh, USA

^4^ Department of Psychiatry and Neurochemistry, Institute of Neuroscience and Physiology, The Sahlgrenska Academy, University of Gothenburg, Mölndal, Sweden

^5^ Wallenberg Centre for Molecular Medicine, University of Gothenburg, Gothenburg, Sweden

^6^ King's College London, Institute of Psychiatry, Psychology and Neuroscience, Maurice Wohl Institute Clinical Neuroscience Institute, London, UK

^7^ NIHR Biomedical Research Centre for Mental Health and Biomedical Research Unit for Dementia at South London and Maudsley NHS Foundation, London, UK

^8^ Clinical Neurochemistry Laboratory, Sahlgrenska University Hospital, Mölndal, Sweden

^9^ Department of Neurodegenerative Disease, UCL Institute of Neurology, Queen Square, London, UK

^10^ UK Dementia Research Institute at UCL, London, UK

^11^ Hong Kong Center for Neurodegenerative Diseases, Clear Water Bay, Hong Kong, China

^12^ Wisconsin Alzheimer’s Disease Research Center, University of Wisconsin School of Medicine and Public Health, University of Wisconsin-Madison, Madison, WI, USA

^13^ Department of Psychiatry, McGill University

**Correspondence author:**

**Pedro Rosa-Neto, MD, PhD***The McGill University Research Centre for Studies in Aging*

*6875 LaSalle Boulevard, Montreal, QC H4H 1R3, Canada*

***E-mail:*** [***pedro.rosa@mcgill.ca***](mailto:pedro.rosa@mcgill.ca)

**Methods. CSF and Plasma Sample Handling and Assays**

CSF samples in the TRIAD cohort were retrieved with lumbar puncture, first using an 18 ga “introducer” to penetrate the interspinous ligaments, followed by dural puncture using the 24 ga Sprotte atraumatic needle. 29 ml of fluid was collected with polypropylene syringes, from which the first 4 mL were sent to a local laboratory for routine analyses. The remaining volume was preserved in polypropylene tubes and centrifuged at 20 degrees Celsius (°C) for 10 minutes at 2200g, after which samples were rapidly frozen for permanent storage at -80°C. Blood samples were collected following previously described protocols [[1]](https://sciwheel.com/work/citation?ids=9168410&pre=&suf=&sa=0&dbf=0). Both CSF and plasma p-tau were quantified in the Clinical Neurochemistry Laboratory, University of Gothenburg by scientists blinded to participant clinical information. CSF concentrations of p-tau_181_ and p-tau_217_ were quantified using a custom single molecule array (Simoa) assay. Plasma p-tau_181_ was measured using an in-house Simoa method (Simoa HD-X instruments, Quanterix, Billerica, MA, USA), as described previously. Plasma p-tau_217_ concentrations were measured using a Simoa assay developed by Janssen [[2]](https://sciwheel.com/work/citation?ids=11706453&pre=&suf=&sa=0&dbf=0). The assay detects phosphorylation at the threonine amino acid at position 217, enhanced by phosphorylation at the threonine amino acid at position 212.

***Supplementary References***

[1. Karikari TK, Pascoal TA, Ashton NJ, Janelidze S, Benedet AL, Rodriguez JL, et al. Blood phosphorylated tau 181 as a biomarker for Alzheimer’s disease: a diagnostic performance and prediction modelling study using data from four prospective cohorts. Lancet Neurol. 2020;19:422–33.](https://sciwheel.com/work/bibliography/9168410)

[2. Triana-Baltzer G, Moughadam S, Slemmon R, Van Kolen K, Theunis C, Mercken M, et al. Development and validation of a high-sensitivity assay for measuring p217+tau in plasma. Alzheimers Dement (Amst). 2021;13:e12204.](https://sciwheel.com/work/bibliography/11706453)

**Supplementary Figure 1. *APOE* modulates the effect of HT on regional Aβ-PET and tau-PET in the post-menopausal females**

Results from voxel-based analyses showed that in post-menopausal HT nonusers, *APOEε4* carriers presented with significantly higher Aβ and NFT load compared to *APOEε4* non-carriers. In contrast, post-menopausal females who use HT showed similar levels of Aβ-PET and tau-PET, regardless of their APOE genotypes. Images represent voxel-based t-statistical parametric maps overlaid on the structural MRI reference template. Age, education and clinical diagnosis were used as covariates in the model. Results were corrected for multiple comparisons using the FDR cluster threshold of *P* < 0.001.

|  | **Postmenopausal HT- female** | **Postmenopausal HT+ female** |
| --- | --- | --- |
| **Amyloid-PET** | **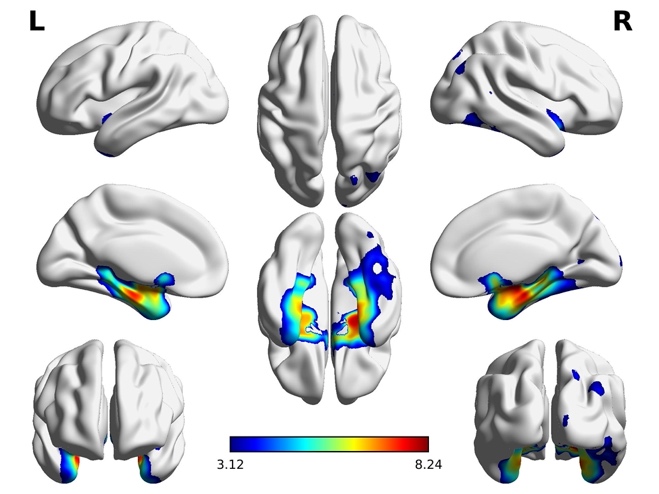**  **E4 > nonE4** | **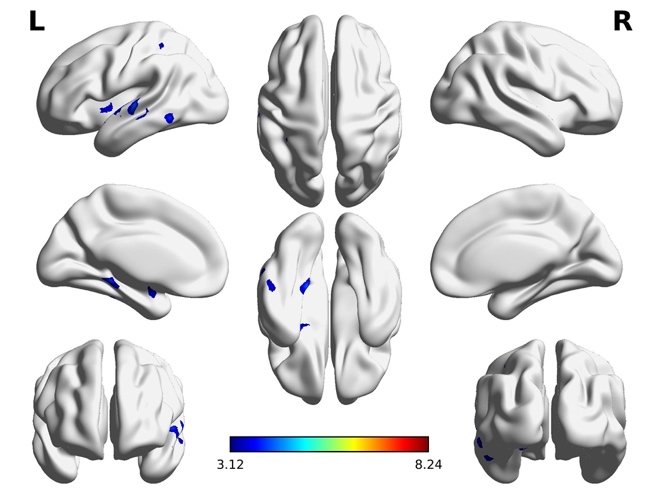**  **E4 > nonE4** |
| **Tau-PET** | **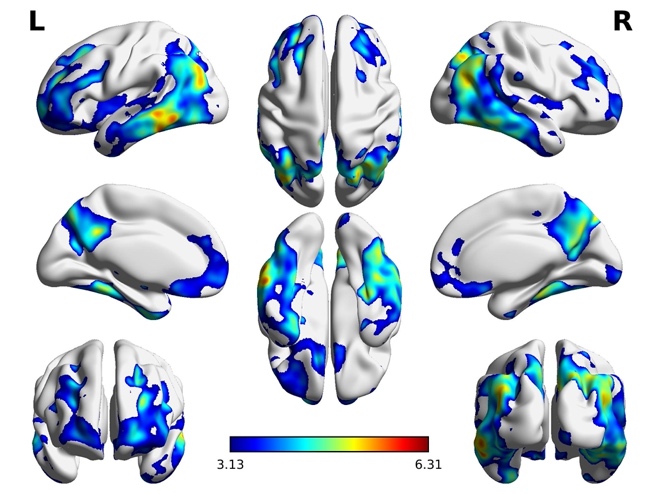**  **E4 > nonE4** | **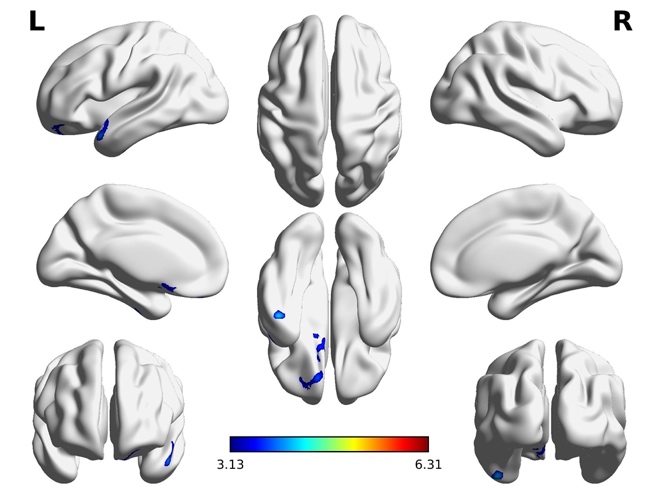**  **E4 > nonE4** |

**Supplementary Figure 2. Hormone therapy interacts with cortical Aβ and is associated with lower regional NFT load**

Linear regression models showed that with similar Aβ load, HT+ females demonstrated less NFT aggregation compared to HT- females in the entorhinal, amygdala, fusiform, hippocampus, parahippocampal, and inferior temporal regions, suggesting HT use interacted with cortical Aβ and mitigated regional NFT load.


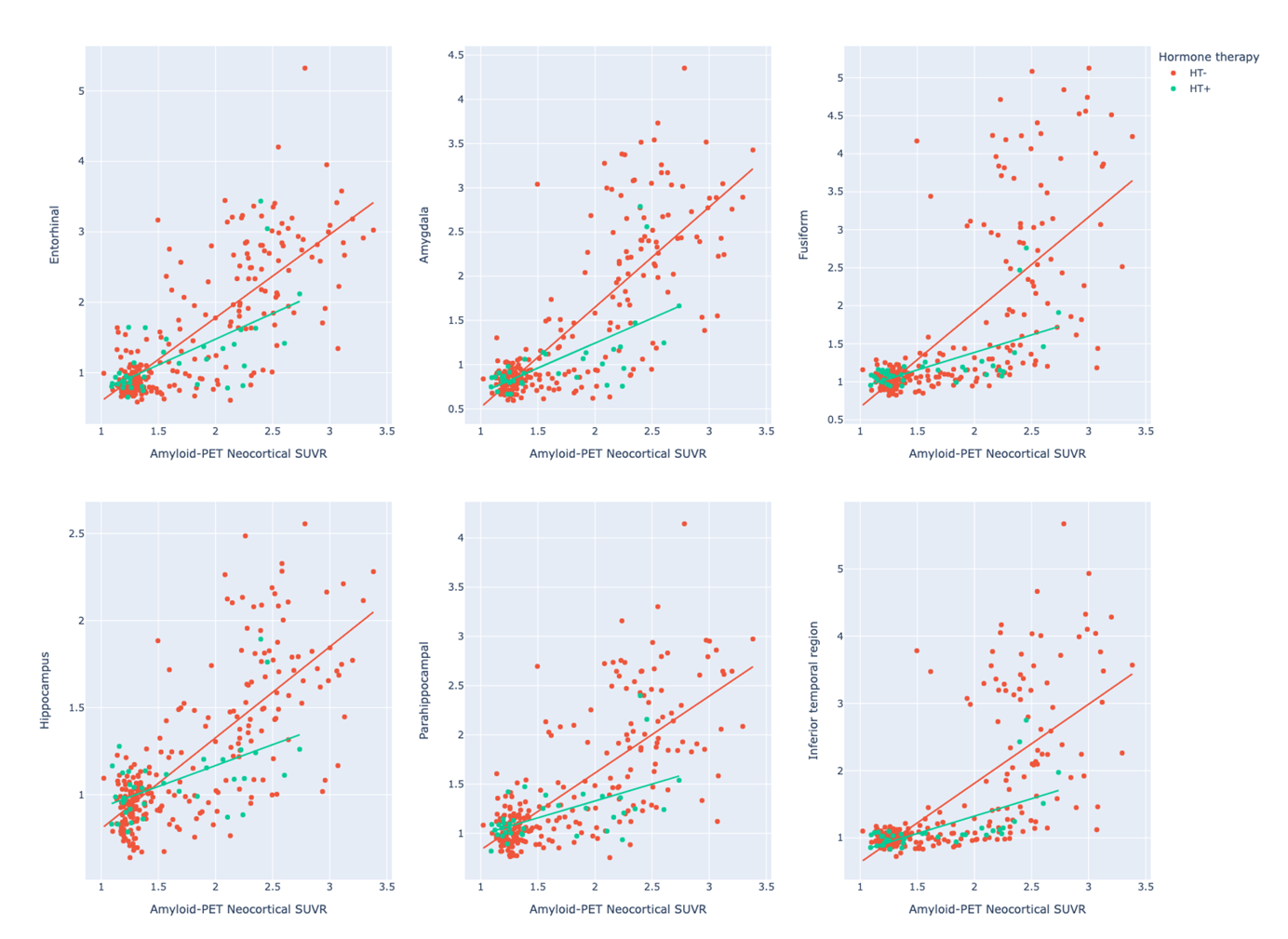


**Supplementary Figure 3. Hormone therapy use is associated with lower p-tau concentrations**

Linear models demonstrated that under similar Aβ-PET SUVR, cognitively impaired (CI) HT+ females presented lower plasma p-tau_181_ and p-tau_217_ concentrations compared to CI HT- females, indicating HT modulated the relationships between Aβ and tau phosphorylation.

|  | **TRIAD** | | **ADNI Fluid** |
| --- | --- | --- | --- |
| **CSF p-tau** | **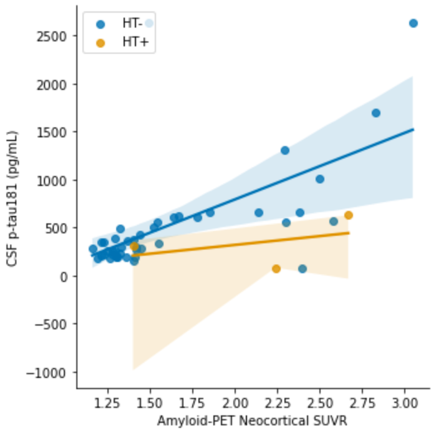** | **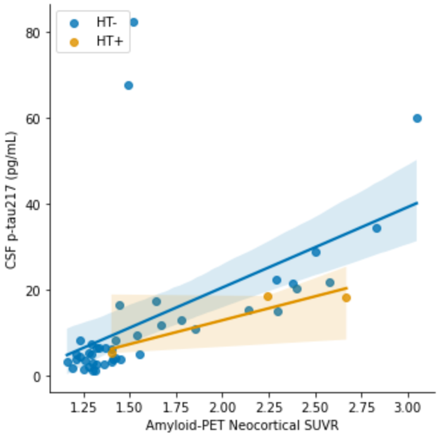** | **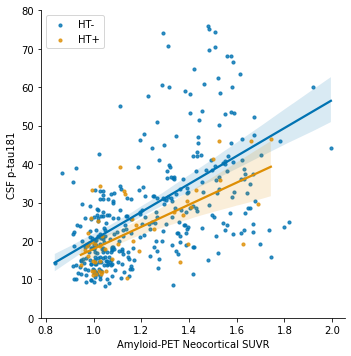** |
| **Plasma p-tau** | **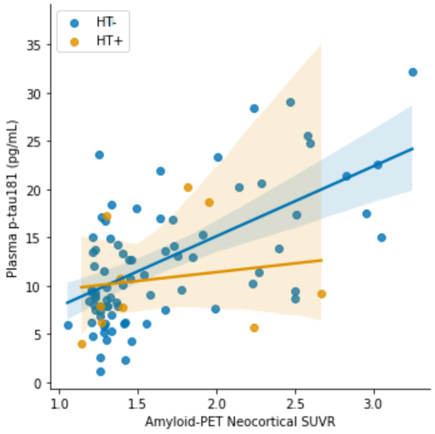** | **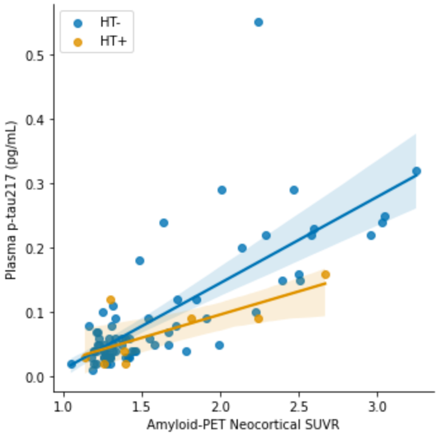** | **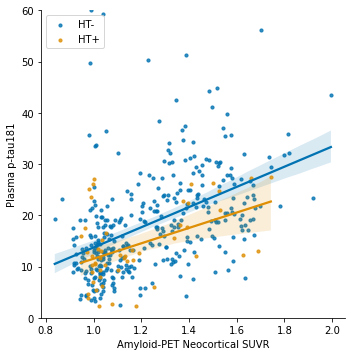** |
